# Fine-grained recognition of plants from images

**DOI:** 10.1186/s13007-017-0265-4

**Published:** 2017-12-21

**Authors:** Milan Šulc, Jiří Matas

**Affiliations:** Department of Cybernetics, FEE CTU in Prague, Karlovo namesti 13, 121 35 Prague 2, Czech Republic

**Keywords:** Computer vision, Plants, Leaves, Bark, Texture, Deep learning, Convolutional neural networks, SVM, Kernel maps

## Abstract

**Background:**

Fine-grained recognition of plants from images is a challenging computer vision task, due to the diverse appearance and complex structure of plants, high intra-class variability and small inter-class differences. We review the state-of-the-art and discuss plant recognition tasks, from identification of plants from specific plant organs to general plant recognition “in the wild”.

**Results:**

We propose texture analysis and deep learning methods for different plant recognition tasks. The methods are evaluated and compared them to the state-of-the-art. Texture analysis is only applied to images with unambiguous segmentation (bark and leaf recognition), whereas CNNs are only applied when sufficiently large datasets are available. The results provide an insight in the complexity of different plant recognition tasks. The proposed methods outperform the state-of-the-art in leaf and bark classification and achieve very competitive results in plant recognition “in the wild”.

**Conclusions:**

The results suggest that recognition of segmented leaves is practically a solved problem, when high volumes of training data are available. The generality and higher capacity of state-of-the-art CNNs makes them suitable for plant recognition “in the wild” where the views on plant organs or plants vary significantly and the difficulty is increased by occlusions and background clutter.

## Background

Recognition of natural objects in the surrounding environment has been of great importance for the humankind since time immemorial. The desire to understand and describe the living nature lead scientists to create systems of biological classification, counting an enormous number of categories and species. For illustration: while the 10th edition of Linnaeus’s Systema Naturae [1] describes about 6000 plant species [2], currently the number of published and accepted plant species in the world is over 310,000 [3].

We study and develop computer vision algorithms to assist or fully automate the plant identification process. From the machine learning point of view, plant recognition is a fine-grained classification task with high intra-class variability and often small inter-class differences, which are often related to the taxonomic hierarchical classification.

Computer vision methods for plant recognition have a number of applications, including mobile field guides using computer vision to automate or speed up the identification process, image data processing for biological databases, automatic detection, registration and mapping of plants from publicly available data, automation in agriculture, etc.

The rest of this section contains a review of the state-of-the art in plant recognition and in the related computer vision areas—texture recognition and deep learning. Our previously published methods and experiments [4–8], on which this article is based, are not mentioned in this section but rather described in more detail, extended and discussed in the rest of the article.

### Plant recognition

Interest in methods for visual classification of plants has grown recently [9–12] as devices equipped with cameras became ubiquitous, making intelligent field guides, education tools and automation in forestry and agriculture practical. Belhumeur et al. [9] discuss the use of such a system in the field allowing a botanist to quickly search entire collections of plant species—a process that previously took hours can now be done in seconds. Plant recognition has been posed, almost without exceptions [13, 14], as recognition of photos depicting solely a specific plant organ such as flower, bark, fruit, leaf or their combination [9–12, 15–27].

#### Leaf recognition

Leaf recognition has been by far the most popular approach to plant recognition and a wide range of methods has been reported in the literature [9, 11, 12, 15–27]. Recognition of leaves usually refers only to recognition of broad leaves, needles are treated separately. Several techniques have been proposed for leaf description, often based on combining features of different character (shape features, colour features, etc.).

A bag of words model with Scale Invariant Feature Transform (SIFT [28]) descriptors was applied to leaf recognition by Fiel and Sablatnig [11]. Several shape methods have been compared on leaf recognition by Kadir et al. [15]. Of the compared methods—geometric features, moment invariants, Zernike moments and polar Fourier Transform—the last performed best on an unpublished dataset.

Kumar et al. [12] describe Leafsnap,^1^ a computer vision system for automatic plant species identification, which has been developed from the earlier plant identification system by Agarwal et al. [16] and Belhumeur et al. [9]. Kumar et al. [12] introduced a pre-filter on input images, numerous speed-ups and additional post-processing within the segmentation algorithm, the use of a simpler and more efficient curvature-based recognition algorithm. On the introduced Leafsnap database of 184 tree species, their recognition system finds correct matches among the top 5 results for 96.8% queries from the dataset. The resulting electronic Leafsnap field guide is available as a mobile app for iOS devices. The leaf images are processed on a server, internet connection is thus required for recognition, which may cause problems in natural areas with slow or no data connection. Another limit is the need to take the photos of the leaves on a white background.

Wu et al. [17] proposed a probabilistic neural network for leaf recognition using 12 digital morphological features, derived from 5 basic features (diameter, physiological length, physiological width, leaf area, leaf perimeter). The authors collected a publicly available plant leaf database named Flavia.

Kadir et al. [24] prepared the Foliage dataset, consisting of 60 classes of leaves, each containing 120 images. The best reported result on this dataset reported by Kadir et al. [18] was achieved by a combination of shape, vein, texture and colour features processed by principal component analysis before classification by a probabilistic neural network.

Söderkvist [25] proposed a visual classification system of leaves and collected the so called Swedish dataset containing scanned images of 15 classes of Swedish trees. Qi et al. [29] achieve 99.38% accuracy on the Swedish dataset using a texture descriptor called Pairwise Rotation Invariant Co-occurrence Local Binary Patterns [27] with Support Vector Machine (SVM) classification.

Novotný and Suk [22] proposed a leaf recognition system, using Fourier descriptors of the leaf contour normalised to translation, rotation, scaling and starting point of the boundary. The authors also collected a large leaf dataset called Middle European Woods (MEW) containing 153 classes of native or frequently cultivated trees and shrubs in Central Europe. Their method achieves 84.92% accuracy when the dataset is split into equally sized training and test set. MEW and Leafsnap are the most challenging leaf recognition datasets.

One possible application of leaf description is the identification of a disease. Pydipati et al. [30] proposed a system for citrus disease identification using color co-occurrence method (CCM), achieving accuracies of over 95% for 4 classes (normal leaf samples and samples with a greasy spot, melanose, and scab).

#### Tree bark recognition

The problem of automatic tree identification from photos of bark can be naturally formulated as texture recognition.

Several methods have been proposed and evaluated on datasets which are not publicly available. Chi et al. [31] proposed a method using Gabor filter banks. Wan et al.[32] performed a comparative study of bark texture features: the grey level run-length method, co-occurrence matrices method, histogram method and auto-correlation method. The authors also show that the performance of all classifiers improved significantly when color information was added. Song et al. [33] presented a feature-based method for bark recognition using a combination of Grey-Level Co-occurrence Matrix (GLCM) and a binary texture feature called long connection length emphasis. Huang et al. [34] used GLCM together with fractal dimension features for bark description. The classification was performed by artificial neural networks.

Since the image data used in the experiments discussed above is not available, it is difficult to assess the quality of the results and to perform comparative evaluation.

Fiel and Sablatnig [11] proposed methods for automated identification of tree species from images of the bark, leaves and needles. For bark description they created a Bag of Words with SIFT descriptors in combination with GLCM and wavelet features. SVM with radial basis function kernel was used for classification. They introduced the Österreichische Bundesforste AG (Austrian Federal Forests) bark dataset consisting of 1182 photos from 11 classes. We refer to this dataset as the AFF bark dataset. A recognition accuracy of 64.2 and 69.7% was achieved on this dataset for training sets with 15 and 30 images per class.

Fiel and Sablatnig also describe an experiment with two human experts, a biologist and a forest ranger, both employees of Österreichische Bundesforste AG. Their classification rate on a subset of the dataset with 9 images per class, 99 images in total, was 56.6% (biologist) and 77.8% (forest ranger).

Boudra et al. [35] review and compare different variants of multi-scale Local Binary Patterns based texture descriptors and evaluate their performance in tree bark image retrieval.

#### Plant identification from diverse images

Recognition of plants given several images of different content-types, such as different plant organs or the entire plant, should be in principle more reliable than recognition only given a one image of one specific plant organ such as leaf or bark. On the other hand, the task is more challenging if an image of an unspecified organ is given. Such problems are posed by the Plant Identification task of the LifeCLEF workshop [14, 36, 37], known as the PlantCLEF challenge, since 2014. The challenge tasks have slightly changed every year. Our contributions to the 2016 and 2017 challenges will be described later in this article.

The 2016 [38] edition of PlantCLEF was evaluated as an *open-set* recognition problem, i.e. “a problem in which the recognition system has to be robust to unknown and never seen categories”. Each image in the task belongs to one of the 7 content-types: leaf, leaf scan, flower, fruit, stem, branch, or entire plant. Albeit the content-type is available in the meta-data, similarly to last years, the best scoring results use the same deep networks for all types of content [39–41]. Ge et al. [42] showed that in this task generic Convolutional Neural Network (CNN) features perform better than content-specific CNN features, and that their combination improves the accuracy. Choi et al. [41] showed that bagging of several generic CNNs improves the accuracy as well, winning the PlantCLEF 2015 challenge.

PlantCLEF 2017 [43] addressed a practical problem of training a very fine grained classifier (10,000 species) from data with noisy labels: Besides 256 thousand labelled images in the “trusted” training set, the organizers also provided URLs to more than 1.4 million weakly-labelled web images in the “noisy” training set, obtained by Google and Bing image search. The evaluation of the task is performed on a test set containing 25,170 images of 13,471 observations (specimen).

Pl@ntNet [13] is another content-type based plant recognition system. It is also an collaborative information system providing an image sharing and retrieval application for plant identification. It has been developed by scientists from four French research organizations (Cirad, INRA, INRIA and IRD) and the Tela Botanica network. The Pl@ntNet-identify Tree Database provides identification by combining information from images of the habitat, flower, fruit, leaf and bark. The exact algorithms used in the Pl@ntNet-identify web service [44] and their accuracies are not publicly documented. There is also a Pl@ntNet mobile app [45], an image sharing and retrieval application for the identification of plants.

### Texture recognition

Texture information is an essential feature for recognition of many plant organs. Texture analysis is a well-established problem with a large number of existing methods, many of them being described in surveys [46–49]. Texture itself is hard to define. There are various definitions of visual texture, but they often lack formality and completeness. For illustration, let us quote an informal definition by Hawkins [50]:

#### **Definition 1**

The notion of texture appears to depend upon three ingredients: (1) some local “order” is repeated over a region which is large in comparison the the order’s size, (2) the order consists in the non-random arrangement of elementary parts, and (3) the parts are roughly uniform entities having approximately the same dimensions everywhere within the textured region.

Here we only review the recent development and the state-of-the-art.

Several recent approaches to texture recognition report excellent results on standard datasets, many of them working only with image intensity and ignoring the available color information. A number of approaches is based on the popular local binary patterns (LBP) [51, 52], such as the recent Pairwise Rotation Invariant Co-occurrence Local Binary Patterns of Qi et al. [27] or the Histogram Fourier Features of Ahonen et al. [53, 54]. A cascade of invariants computed by scattering transforms was proposed by Sifre and Mallat [55] in order to construct an affine invariant texture representation. Mao et al. [56] use a bag-of-words model with a dictionary of so called active patches: raw intensity patches that undergo further spatial transformations and adjust themselves to best match the image regions. While the Active Patch Model doesn’t use color information, the authors claim that adding color will further improve the results. The method of Cimpoi et al. [57] using Improved Fisher Vectors (IFV) for texture description shows further improvement when combined with describable texture attributes learned on the Describable Textures Dataset (DTD) and with color attributes.

Recently, Cimpoi et al. [58, 59] pushed the state-of-the-art in texture recognition using a new encoder denoted as FV-CNN-VD, obtained by Fisher Vector pooling of a very deep convolutional neural network (CNN) filter bank pre-trained on ImageNet by Simonyan and Zisserman [60]. The CNN filter bank operates conventionally on preprocessed RGB images. This approach achieves state-of-the-art accuracy, yet due to the size of the very deep VGG networks it may not be suitable for real-time applications when evaluated without a high-performance graphics processing unit (GPU) for massive parallelization.

### Deep convolutional neural networks

Deep convolutional neural networks (CNNs) succeeded in a number of computer vision tasks, especially those related to complex recognition and detection of objects with large databases of training images, such as the computer vision challenges ImageNet [61], Pascal VOC [62] and Common Objects in Context (COCO) [63]. Since the success of Krizhevsky’s network [64] in the ImageNet 2012 Image Classification challenge, deep learning research leads to state-of-the-art results in such tasks. This was also the case of the PlantCLEF challenges [37, 38, 43], where the deep learning submissions [41, 42, 65, 66] outperformed combinations of hand-crafted methods significantly.

Recently, the very deep residual networks of He et al. [67] gained a lot of attention after achieving the best results in both the ILSVRC (ImageNet Large Scale Visual Recognition Challenge) 2015 and the COCO 2015 Detection Challenge. The residual learning framework allows to efficiently train networks that are substantially deeper than the previously used CNN architectures.

Szegedy et al. [68] study the ways to scale up networks efficiently by factorized convolutions and aggressive regularization. Their study is performed on Inception-style networks (i.e. networks with architectures similar to GoogleNet [69]), and propose the so called Inception v3 architecture. Furthermore, Szegedy et al. [70] show that training with residual connections accelerates the training of Inception networks significantly and that a residual Inception networks may outperform a similarly expensive Inception networks without residual connections by a thin margin.

## Methods

### Texture recognition approach to plant identification

Inspired by the textural nature of bark and leaf surfaces, we approach plant recognition as texture classification. In order to describe texture independently of the pattern size and orientation in the image, a description invariant to rotation and scale is needed. For practical applications we also demand computational efficiency.

We introduce novel texture description called *Fast Features Invariant to Rotation and Scale of Texture (Ffirst)*, which combines several design choices to satisfy the given requirements. This method builds on and improves our texture descriptor for bark recognition [4].

#### Completed local binary pattern and histogram fourier features

The Ffirst description is based on the Local Binary Patterns [51, 52, 71]. The common LBP operator (later denoted as sign-LBP) locally computes the signs of differences between the center pixel and its *P* neighbours on a circle of radius *R*. With an image function *f*(*x*, *y*) and neighbourhood point coordinates $(x_p,y_p)$:

1 $$\begin{aligned} \begin{aligned} \text {LBP}_{P,R} (x,y)&= \sum \limits _{p=0}^{P-1} s( f(x,y) - f(x_p,y_p) ) 2^p , \; s(z)&=\left\{ \begin{array}{ll} 1 : &{} \text {if } z \le 0,\\ 0 : &{} \text {otherwise.} \end{array} \right. \end{aligned} \end{aligned}$$

To achieve rotation invariance,^2^ we adopt the so called LBP histogram Fourier features (LBP-HF) introduced by Ahonen et al. [53]. LBP-HF describe the histogram of uniform patterns using coefficients of the discrete Fourier transform (DFT). Uniform LBP are patterns with at most 2 spatial transitions (bitwise 0-1 changes). Unlike the simple rotation invariants using $\hbox {LBP}^\text {ri}$ [71, 72], which joins all uniform patterns with the same number of 1s into one bin, the LBP-HF features preserve the information about relative rotation of the patterns.

Denoting a uniform pattern $U_p ^{n,r}$, where *n* is the “orbit” number corresponding to the number of “1” bits and *r* denotes the rotation of the pattern, the DFT for given *n* is expressed as:

2 $$\begin{aligned} H(n,u) = \sum \limits _{r=0}^{P-1} h_I\left( U_p^{n,r}\right) e^{-i2\pi u r /P} \,, \end{aligned}$$

where the histogram value $h_I (U_p^{n,r})$ denotes the number of occurrences of a given uniform pattern in the image.

The LBP-HF features are equal to the absolute value of the DFT magnitudes, and thus are not influenced by the phase shift caused by rotation).

3 $$\begin{aligned} {LBP-HF}(n,u) = \vert H(n,u) \vert = =\sqrt{ H(n,u) \overline{H(n,u)}} . \end{aligned}$$

Since $h_I$ are real, $H(n,u) = H(n,P-u)$ for $u = (1,\ldots ,P-1)$, and therefore only $\left\lfloor {\frac{P}{2}}\right\rfloor +1$ of the DFT magnitudes are used for each set of uniform patterns with *n* “1” bits for $0<n<P$. Three other bins are added to the resulting representation, namely two for the “1-uniform” patterns (with all bins of the same value) and one for all non-uniform patterns.

The LBP histogram Fourier features can be generalized to any set of uniform patterns. In Ffirst, the LBP-HF-S-M description [54] is used, where the histogram Fourier features of both sign- and magnitude-LBP are calculated to build the descriptor. The magnitude-LBP [73] checks if the magnitude of the difference of the neighbouring pixel $(x_p,y_p)$ against the central pixel (*x*, *y*) exceeds a threshold $t_p$:

4 $$\begin{aligned} \text {LBP-M}_{P,R} (x,y) = \sum _{p=0}^{P-1} s( \vert f(x,y) - f(x_p,y_p) \vert - t_p) 2^p . \end{aligned}$$

We adopted the common practice of choosing the threshold value (for neighbours at *p*-th bit) as the mean value of all *m* absolute differences in the whole image:

5 $$\begin{aligned} t_p = \sum \limits _{i=1}^m \dfrac{ \vert f(x_i,y_i) - f(x_{ip},y_{ip}) \vert }{m}. \end{aligned}$$

The LBP-HF-S-M histogram is created by concatenating histograms of LBP-HF-S and LBP-HF-M (computed from uniform sign-LBP and magnitude-LBP).

#### Multi-scale description and scale invariance

A scale space is built by computing LBP-HF-S-M from circular neighbourhoods with exponentially growing radius *R*. Gaussian filtering is used^3^ to overcome noise.

Unlike the MS-LBP approach of Mäenpää and Pietikäinen [74], where the radii of the LBP operators are chosen so that the effective areas of different scales touch each other, Ffirst uses a finer scaling with a step of $\sqrt{2}$ between scales radii $R_i$, i.e. $R_i = R_{i-1} \sqrt{2}$. This radius change is equivalent to decreasing the image area to one half. The first LBP radius used is $R_1=1$, as the LBP with low radii capture important high frequency texture characteristics.

Similarly to [74], the filters are designed so that most of their mass lies within an effective area of radius $r_i$. We select the effective area diameter, such that the effective areas at the same scale touch each other: $r_i = R_i \sin \frac{\pi }{P}$.

**Fig. 1:**
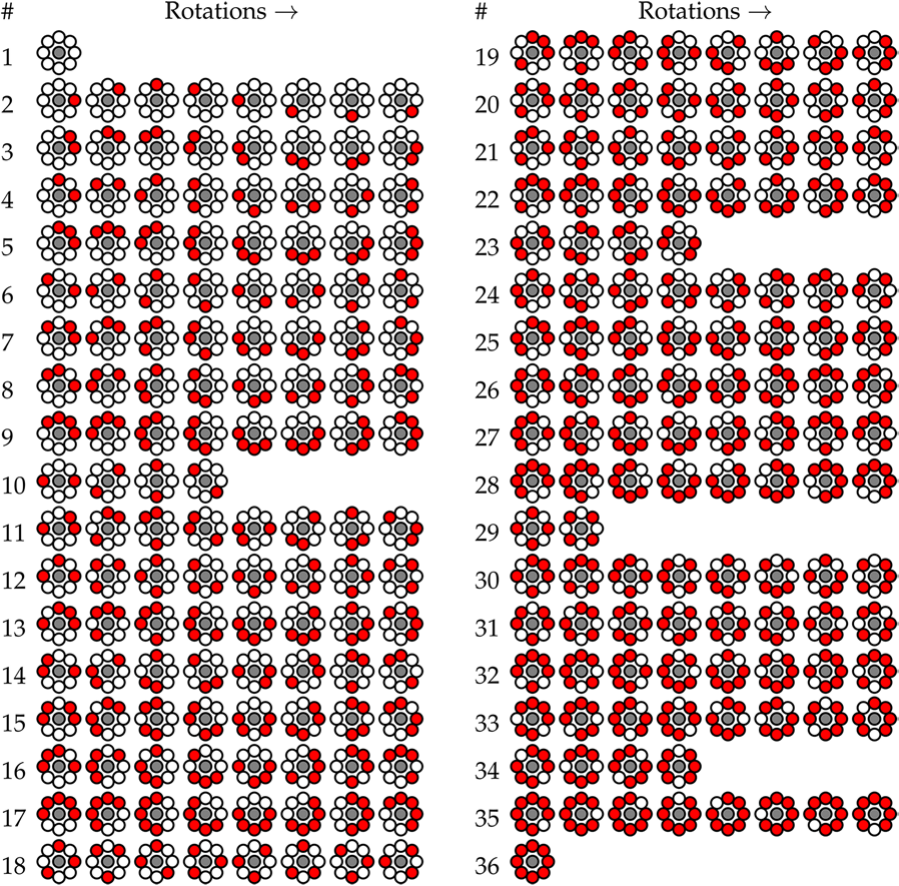
The full set of local binary patterns divided into 36 orbits for the Histogram Fourier features. Patterns in one orbit only differ by rotation

LBP-HF-S-M histograms from *c* adjacent scales are concatenated into a single descriptor. Invariance to scale changes is increased by creating $n_\text {conc}$ multi-scale descriptors for one image. See Fig. 1 for the overview of the texture description method.

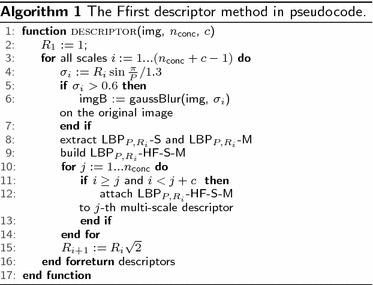



#### Support Vector Machine and feature maps

In most applications, a Support Vector Machine (SVM) classifier with a suitable non-linear kernel provides higher recognition accuracy at the price of significantly higher time complexity and higher storage demands (dependent on the number of support vectors). An approach for efficient use of additive kernels via explicit feature maps is described by Vedaldi and Zisserman [75] and can be combined with a linear SVM classifier. Using linear SVMs on feature-mapped data improves the recognition accuracy, while preserving linear SVM advantages like fast evaluation and low storage (independent on the number of support vectors), which are both very practical in real time applications. In Ffirst we use the explicit feature map approximation of the histogram intersection kernel, although the $\chi ^2$ kernel leads to similar results.

The "One versus All" classification scheme is used for multi-class classification, implementing the Platt’s probabilistic output [76, 77] to ensure SVM results comparability among classes. The maximal posterior probability estimate over all scales is used to determine the resulting class.

In our experiments we use a stochastic dual coordinate ascent [78] linear SVM solver implemented in the VLFeat library [79].

#### Adding rotational invariants

The LBP-HF features used in the proposed Ffirst description are usually built from the DFT magnitudes of differently rotated uniform patterns. We propose to use all LBP instead of just the subset of uniform patterns. Note that in this case, some orbits have a lower number of patterns, since some non-uniform patterns show symmetries, as illustrated in Fig. 1.

Another rotational invariants are computed from the first DFT coefficients for each orbit:

6 $$\begin{aligned} \text {LBP-HF}^{+}(n) = \sqrt{ H(n,1) \overline{H(n+1,1)}} \end{aligned}$$

$\hbox {Ffirst}^{\forall +}$ denotes the method using the full set of patterns for LBP-HF features and adding the additional LBP-$\hbox {HF}^{+}$ features.

#### Recognition of segmented textural objects

We propose to extend Ffirst to segmented textural objects by treating the border and the interior of the object segment separately.

**Fig. 2:**
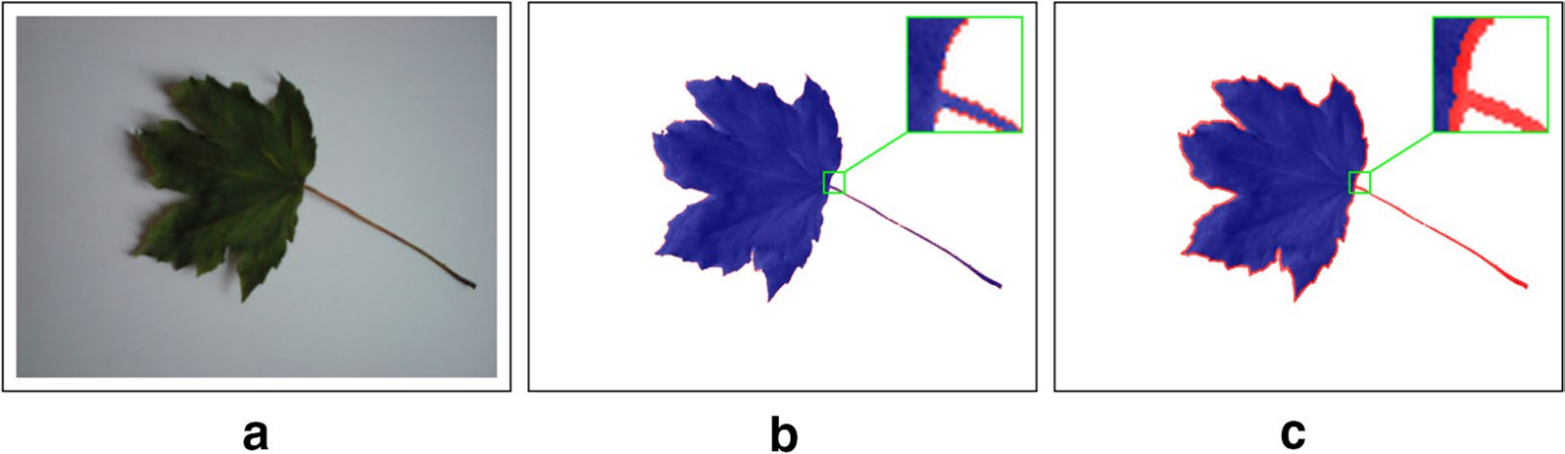
Segmentation of the leaf interior (blue) and border region (red) at different scales given by LBP radius *R*. The border region is defined as all points which have at least one neighbour (in $\mathrm{LBP}_{P,R}$) outside of the segmented region.** a** Original image,** b** Segmentation, R = 2.8,** c** Segmentation, R = 11.3

Let us consider a segmented object region ${\mathbb {A}}$. One may describe only points that have all neighbours at given scale inside ${\mathbb {A}}$. We show that describing a correctly segmented border, i.e. points in ${\mathbb {A}}$ with one or more neighbours outside ${\mathbb {A}}$ (see Fig. 2), adds additional discriminative information.

We experiment with 5 variants of the recognition method, differing in the processing of the border region:

1. $\hbox {Ffirst}_\text {a}$ describes all pixels in ${\mathbb {A}}$ and maximizes the posterior probability estimate (i.e. SVM Platt’s probabilistic output) over all $n_\text {conc}$ scales.
2. $\hbox {Ffirst}_\text {i}$ describes only the segment interior, i.e. pixels in ${\mathbb {A}}$ with all neighbours in ${\mathbb {A}}$.
3. $\hbox {Ffirst}_\text {b}$ describes only the segment border, i.e. pixels in ${\mathbb {A}}$ with at least one neighbour outside ${\mathbb {A}}$.
4. $\hbox {Ffirst}_{\text {ib}{\sum }}$ combines the $\hbox {Ffirst}_\text {i}$ and $\hbox {Ffirst}_\text {b}$ descriptors and maximizes the sum of their posterior probability estimates over $n_\text {conc}$ scales.
5. $\hbox {Ffirst}_{\text {ib}{\prod }}$ combines the $\hbox {Ffirst}_\text {i}$ and $\hbox {Ffirst}_\text {b}$ descriptors and maximizes the product of their posterior probability estimates over $n_\text {conc}$ scales.

The leaf databases contain images of leaves on an almost white background. Segmentations were obtained by thresholding using the Otsu’s method [80].

### Deep learning approach to plant identification

For significantly more complex tasks—where the photos are nearly unconstrained (depicting different plant organs or the whole plant in its natural environment), with complex background, and much higher numbers of classes (10,000 in the case of LifeCLEF 2017 [81]), we choose a deep learning approach and utilize state-of-the-art deep convolutional neural networks, which succeeded in a number of computer vision tasks, especially those related to complex recognition and detection of objects. Given the enormous popularity of convolutional neural networks in the last years and the volume of available deep learning literature (e.g. [82–84]), we skip most of the deep learning theory and we only briefly describe our choices of architectures, models and techniques for our contributions to the PlantCLEF challenges.

In the experiments, we used the state-of-the-art CNN architectures as a baseline and added modifications described below: ensemble training with bagging, maxout, and bootstrapping for training on noisy labels. We initialized all convolutional layer parameters from networks pre-trained on the 1 million ImageNet images, and then fine-tuned the networks on the training data for the plant recognition task. Such initialization is a common practice that speeds up training and helps to avoid early overfitting on tasks with a small number of training images.

#### Bagging

In deep learning challenges it is a common practice to train several networks on different (but not necessarily mutually exclusive) subsets of the training data. An ensemble of such networks, commonly combined by a simple voting mechanism (e.g. sum or maximum of class prediction scores), tends to outperform individual networks. In the PlantCLEF 2015 plant classification challenge, Choi [41] gained a significant margin in precision using bagging of 5 networks.

#### Maxout

Maxout [85] is based on an activation function, which takes a maximum over *k* parts (e.g. slices) of a network layer:

7 $$\begin{aligned} h_i(x)=\max _{j\in \left[ 1,k\right] } z_{ij} , \end{aligned}$$

where $z_{ij} = {\mathbf {x}}^\text {T}{\mathbf {W}}_{..ij} + b_{ij}$ can be a standard fully connected (FC) layer with parameters $W \in {\mathbb {R}}^{d\times m \times k}$, $b \in {\mathbb {b}}^ {m \times k}$.

One can understand maxout as a piecewise linear approximation to a convex function, specified by the weights of the previous layer. Maxout was designed [85] to be combined with dropout [86].

The maxout is not used on top of the FC classification layer (which would mean increasing its size *k*-times), we add an additional FC layer with maxout activation before the classification FC layer.

#### Bootstrapping

In order to improve learning from noisy labels in the scenario of the PlantCLEF 2017 plant identification challenge, we experimented with the so called “bootstrapping” of Reed et. al. [87]. An objective is proposed that takes into account the current predictions of the network, with the intention to lower the effect of incorrect labels. Reed et al. propose two variants of the objective:

- *Soft bootstrapping* uses the probabilities $q_k$ given by the network (softmax):
  8 $$\begin{aligned} { L }_\text {soft} ({\mathbf {q}},{\mathbf {t}}) = \sum _{k=1}^N \left[ \beta t_k + ( 1 - \beta ) q_k \right] \log q_k, \end{aligned}$$
  where $t_k$ are the provided labels and $\beta$ is a parameter of the method. The authors [87] point out that the objective is equivalent to softmax regression with minimum entropy regularization, which was previously studied in [88]; encouraging high confidence in predicting labels.
- *Hard bootstrapping* uses the strongest prediction $$z_k = {\left\{ \begin{array}{ll}1 \text { if } k=\text {argmax}q_i \\ 0 \text { otherwise}\end{array}\right. }$$
  9 $$\begin{aligned} { L }_\text {hard} ({\mathbf {q}},{\mathbf {t}}) = \sum _{k=1}^N \left[ \beta t_k + ( 1 - \beta ) z_k \right] \log q_k \end{aligned}$$

We decided to follow the best performing setting of [87] and use hard booststrapping with $\beta =0.8$ in our experiments. The search for the optimal value of $\beta$ was omitted for computational reasons and limited time for the competition, yet the dependence between the amount of label noise and the optimal setting of hyperparameter $\beta$ is a topic for future work.

#### ResNet with maxout for LifeCLEF 2016

In LifeCLEF 2016, we utilized the state-of-the-art very deep 152-layer residual network of He et al. [67]. The residual learning framework allows to efficiently train networks that are substantially deeper than the previously used CNN architectures. We used the model pre-trained on ImageNet which is publicly available [89] and inserted an additional fully connected layer sliced into 4 parts with 512 neurons each, and applied the maxout activation function on the slices. The parameters of both the new FC layer and the following 1000-way FC classification layer were initialized using the method of Glorot [90].

Thereafter, we fine-tuned the network for 150,000 iterations with the following parameters:

- The learning rate was set to $10^{-3}$ and lowered by a factor of 10 after every 100,000 iterations.
- The momentum was set to 0.9, weight decay to $2\cdot 10^{-4}$. r
- The effective batch size was set to 28 (either computed at once on NVIDIA Titan X, or split into more batches using Caffe’s *iter_size* parameter when used on GPUs with lower VRAM).
- A horizontal mirroring of input images was performed during training.

Due to computational limits at training time, we only performed bagging of 3 networks, despite we expect that using a higher number of bagged networks would further improve the accuracy. For training the ensemble of networks, a different $\frac{1}{3}$ of the training data was removed in each bag. The voting was done by taking species-wise maximum of output probabilities.

#### Inception-ResNet-v2 with maxout for LifeCLEF 2017

Our model for PlantCLEF 2017 was based on the state-of-the-art convolutional neural network architecture, the Inception-ResNet-v2 model [70], which introduced residual Inception blocks - a new type of the Inception block making use of the residual connections from [67]. Both the paper [70] and our preliminary experiments show that this network architecture leads to results superior to other state-of-the-art CNN architectures. The publicly available [91] Tensorflow model pretrained on ImageNet was used to initiate the parameters of convolutional layers. The main hyperparameters were set as follows:

- Optimizer: RMSProp with momentum 0.9 and decay 0.9.
- Weight decay: 0.00004.
- Learning rate: Starting LR 0.01 with decay factor 0.94, exponential decay, ending LR 0.0001.
- Batch size: 32.

We added a FC layer with 4096 units. The maxout activation operates over $k=4$ linear pieces the FC layer, i.e. $m=1024$. Dropout with a keep probability of 80% is applied before the FC layers. The final layer is a 10,000-way softmax classifier corresponding to the number of plant species needed in the 2017 task.

The PlantCLEF 2017 training data consists of 2 sets, both covering the same 10,000 plant species:

1. A “trusted” training set based on the online collaborative Encyclopedia Of Life (EoL), where the ground truth labels should be assigned correctly.
2. The “noisy” training set built using web crawlers (more precisely, the Google and Bing image search results) and may thus contain images which are not related to the declared plant species.

We fine-tuned our networks in three different ways:

1. Using only “trusted” (EoL) training data.
2. Using both “trusted” and “noisy” training data (EoL + web).
3. Filtering the “noisy” data using a model pretrained on the “trusted” data, and then fine-tuning on the combination of “trusted” and “filtered noisy” data (EoL + filtered web).

### Datasets and evaluation methodology

Bark recognition is evaluated on a dataset collected by *Österreichische Bundesforste—Austrian Federal Forests*, which was introduced in 2010 by Fiel and Sablatnig [92] and contains 1182 bark images from 11 classes. We denote it as *the Austrian Federal Forests (AFF) bark dataset*.^4^ The resolution of the images varies (between 0.4 and 8.0 Mpx). This dataset is not publicly available, but it was kindly provided by the Computer Vision Lab, TU Vienna, for academic purposes, with courtesy by Österreichische Bundesforste/Archiv.

Unlike in bark recognition, there is a number of existing datasets for leaf classification, most of them being publicly available. The datasets and their experimental settings are briefly described bellow:

*The Austrian Federal Forest (AFF) leaf dataset* was used by Fiel and Sablatnig [11] for recognition of trees, and was kindly provided together with the bark dataset described previously. It contains 134 photos of leaves of the 5 most common Austrian broad leaf trees. The leaves are placed on a white background. The results are compared using the protocol of Fiel and Sablatnig, i.e. using 8 training images per leaf class.

*The Flavia leaf dataset* contains 1907 images (1600 × 1200 px) of leaves from 32 plant species on white background, 50–77 images per class. The dataset was introduced by Wu et al. [17], who used 10 images per class for testing and the rest of the images for training. More recent publications use 10 randomly selected test images and 40 randomly selected training images per class, achieving better recognition accuracy even with the lower number of training samples. In the case of the two best result reported by Lee et al. [20, 21], the number of training samples is not clearly stated.^5^ Some authors divide the set of images for each class into two halves, one for training and the other for testing.

*The Foliage leaf dataset* by Kadir et al. [19, 24] contains 60 classes of leaves from 58 species. The dataset is divided into a training set with 100 images per class and a test set with 20 images per class.

*The Swedish leaf dataset* was introduced in Söderkvist’s diploma thesis [25] and contains images of leaves scanned using a 300 dpi colour scanner. There are 75 images for each of 15 tree classes. The standard evaluation scheme uses 25 images for training and the remaining 50 for testing. Note: The best reported result of Qi et al. [27] was found on the project homepage [29].

*The Leafsnap dataset* version 1.0 by Kumar et al. [12] was publicly released in 2014. It covers 185 tree species from the Northeastern United States. It contains 23147 high quality Lab images and 7719 Field images. The authors note that the released dataset does not exactly match that used to compute results for the paper, nor the currently running version on their servers, yet it seems to be similar to the dataset used in [12] and should allow at least a rough comparison. In the experiments of [12], leave-one-image-out species identification has been performed, using only the Field images as queries, matching against all other images in the recognition database. Probability of the correct match appearing among the top 5 results is taken as the resulting score. Note: The classification accuracy of [12] for the 1st result in Table 2 is estimated from a plot in [12]. Because leave-one-image-out testing scheme would demand to re-train our classifiers for each tested image, we rather perform 10-fold cross validation, i.e. divide the set of Fields images into 10 parts, testing each part on classifiers learned using the set of other parts together with the Lab images.

*The Middle European Woods (MEW) dataset* was introduced by Novotný and Suk [22]. It contains 300 dpi scans of leaves belonging to 153 classes (from 151 botanical species) of Central European trees and shrubs. There are 9745 samples in total, at least 50 per class. The experiments are performed using half of the images in each class for training and the other half for testing.

*The PlantCLEF challenge datasets* depict plants in a significantly wider range of views, such as leaves, flowers, fruits, stems, entire plants and branches.

In the plant identification challenge PlantCLEF 2016, the training set contained 113,205 images of 1000 species of herbs, trees and ferns, and included also other meta-data, such as the type of view (fruit, flower, entire plant, etc.), observation ID and GPS coordinates (if available). The test set contained 8000 pictures, including “distractor” images which did not depict one of the 1000 species.

In the PlantCLEF 2017 challenge, there were two training sets available: a “trusted” set of 256,287 thousand labelled images of 10,000 plant species with meta-data, and a “noisy” set with URLs to more than 1.4 million weakly-labelled web images obtained by Google and Bing image search. The evaluation of the task was performed on a test set containing 25,170 images of 13,471 observations (specimen). There are no “distractor” images in the 2017 test set.

While PlantCLEF 2016 challenge was evaluated based on the mean Average Precision (mAP), PlantCLEF 2017 used a less common measure—the mean reciprocal rank (MRR):

10 $$\begin{aligned} \mathrm{MRR} = \dfrac{1}{\vert Q \vert }\sum ^{\vert Q \vert }_{i=1}\dfrac{1}{\text {rank}_i}, \end{aligned}$$

where $\vert Q \vert$ is the total number of queries in the test set and $\text {rank}_i$ is the rank of the correct result for the *i*-th query.

## Results

### Tree bark classification

Results of our texture recognition approach to tree bark classification on the Austrian Federal Forest bark dataset are compared with the best published results in Table 1. Note that the MS-LBP method assumes the orientation is fixed, which seems to be a useful assumption in the case of this dataset. However, unlike Ffirst, it doesn’t provide rotation invariance. Because the bark dataset is very small, we skip experiments with CNNs, which need a considerably higher amount of data for the standard training/fine-tuning procedures.

**Table 1:** Bark classification results of Ffirst and the state-of-the-art methods

	*AFF* 10 fold	*AFF* 15 train	*AFF* 30 train
$\hbox {Ffirst}^{\forall +}$	96.5 ± 1.2	84.9 ± 2.5	90.4 ± 1.6
MS-LBP-HF [4]	92.2 ± 2.7	74.4 ± 3.4	–
MS-LBP [4]	96.5 ± 2.7	85.5 ± 2.7	–
Fiel, Sablatnig [11, 92]	–	64.2	69.7

Evaluation schemes using 10 fold cross validation, or 15 and 30 training images per class

### Leaf classification

Application of the proposed fast features invariant to rotation and scale of texture to identification of leaves [5] lead to excellent results on standard leaf recognition datasets, proposing a novel approach to visual leaf identification: a leaf is represented by a pair of local feature histograms, one computed from the leaf interior, the other from the border, see Fig. 2. This description utilizing *Ffirst* outperforms the state-of-the-art on all tested leaf datasets—the Austrian Federal Forests dataset, the Flavia dataset, the Foliage dataset, the Swedish dataset and the Middle European Woods dataset—achieving excellent recognition rates above 99%. Updated results of our leaf recognition method originally published in [5] are in Table 2.

Leaf classification with deep convolutional neural networks is hard to apply to experiment with small leaf datasets. To get a comparison with our textural method, we performed our experiment on the Middle European Woods dataset, fine-tuning from an ImageNet-pretrained model. Note that due to high computational complexity and limited GPU resources, we only evaluated this method on one random data split (in both directions), while Ffirst was evaluated on 10 random splits. After 200,000 steps, the Inception-ResNet-v2 network with maxout outperforms previous results significantly, achieving *99.9 and 100.0% accuracy* respectively. Moreover, the correct class always appears among the top 5 predictions.

**Table 2:** Evaluation of Ffirst on available leaf datasets: Austrian Federal Forests, Flavia, Foliage, Swedish, Middle European Woods and Leafsnap

	AFF	Flavia $10\times 40$	Flavia $\frac{1}{2}\times \frac{1}{2}$	Foliage	Swedish	MEW	Leafsnap	Leafsnap top 5
Num. of classes	5	32	32	60	15	153	185	185
$\hbox {Ffirst}_\text {a}^{\forall +}$ (1)	$97.1\pm 1.5$	$99.4\pm 0.3$	$99.2\pm 0.2$	99.2	$99.7\pm 0.3$	$98.8\pm 0.2$	$81.2\pm 1.8$	$95.9\pm 1.5$
$\hbox {Ffirst}_\text {i}^{\forall +}$ (2)	$97.3\pm 1.6$	$99.3\pm 0.3$	$98.9\pm 0.3$	98.1	$99.7\pm 0.3$	$98.4\pm 0.2$	$73.1\pm 2.3$	$92.4\pm 1.7$
$\hbox {Ffirst}_\text {b}^{\forall +}$ (3)	$99.5\pm 0.6$	$99.3\pm 0.4$	$99.0\pm 0.2$	98.3	$99.4\pm 0.5$	$97.9\pm 0.2$	$77.2\pm 1.9$	$94.8\pm 1.5$
$\hbox {Ffirst}_{ib\sum }^{\forall +}$ (4)	$100.0\pm 0.0$	$99.7\pm 0.3$	$99.6\pm 0.1$	99.3	$99.8\pm 0.2$	$99.3\pm 0.1$	$81.8\pm 1.2$	$96.5\pm 1.1$
$Ffirst _{ib\prod }^{\forall +}$ (5)	$100.0 \pm 0.0$	$99.8 \pm 0.3$	99.7 ± 0.1	99.3	$99.8 \pm 0.3$	$99.5 \pm 0.1$	$83.7 \pm 1.1$	$97.3 \pm 1.1$
Inception-ResNet-v2 +maxout	$-$	$-$	$-$	$-$	$-$	99.9+	$-$	$-$
Kumar et al. [12]	$-$	$-$	$-$	$-$	$-$	$-$	$\approx$ 73	96.8
Fiel, Sablatnig [11]	93.6	$-$	$-$	$-$	$-$	$-$	$-$	$-$
Novotný, Suk [22]	$-$	$-$	91.5	$-$	$-$	84.9	$-$	$-$
Karuna et al. [23]	$-$	$-$	96.5	$-$	$-$	$-$	$-$	$-$
Kadir et al. [18]	$-$	95.0	$-$	95.8	$-$	$-$	$-$	$-$
Lee et al. [21]	$-$	97.2	$-$	$-$	$-$	$-$	$-$	$-$
Qi et al. [27]	$-$	$-$	$-$	$-$	99.4	$-$	$-$	$-$

### PlantCLEF plant identification challenges

In the *PlantCLEF 2016* plant identification challenge, our main submission [8] using bagging of our three residual networks with maxout achieved 71.0% mAP (mean average precision), placing us among the top 3 teams in the challenge, where the winning submission achieved 74.2% mAP. Our deep network was actually more precise for single image labelling than the winning submission [39], which pushed the mAP from 61.1 to 74.2% by utilizing the ObservationID meta-information and summing the scores over all images in an observation. Our post-challenge experiments show that summing the scores over observations would boost our system to 78.8% mAP on the PlantCLEF 2016 test data.

For *PlantCLEF 2017*, we fine-tuned our deep networks on the “trusted” (EoL) data only, as well as on the combination of both “trusted” and “noisy” data (EoL + web). We also experimented with the bootstrapping technique for training with “noisy” data. In experiments on our validation set (based on 2016 test data) the networks trained only on the “trusted” data performed slightly better. The two best performing networks trained on the “trusted” (EoL) dataset, each achieving 65% accuracy on the validation set, were then used in the following experiments.

- *Net #1:* Fine-tuned on “trusted” (EoL) set without maxout for 200k it.
- *Net #2:* Fine-tuned on “trusted” (EoL) set with maxout for 200k it.

A “filtered noisy” training set of 425k images was acquire from the noisy set by keeping only images where the prediction of Net #1 was equal to the label.

In order to train ensembles with bagging, we divided the data into 3 disjoint folds. Then the following networks were further fine-tuned on different 2 of the 3 folds for 50,000 iterations.

- *Net #3, #4, #5* Fine-tuned from Net #1 for 50k it. on the “trusted” dataset.
- *Net #6, #7, #8* Fine-tuned from Net #2 for 50k it. on the “trusted” dataset, with maxout.
- *Net #9, #10, #11* Fine-tuned from Net #1 for 50k it. on the “trusted” and “filtered noisy” data.
- *Net #12, #13, #14* Fine-tuned from Net #1 for 50k it. on the “trusted” and “filtered noisy” data, with hard bootstrapping.
- *Net #15,#16,#17* Fine-tuned from Net #2 for 50k it. on the “trusted” and “filtered noisy” data, with maxout.

The individual fine-tuned networks did not achieve much improvement compared to networks #1 and #2: the accuracies ranged from 57 to 67% on the validation set. However combinations of the differently fine-tuned networks are beneficial: an ensemble of all 17 networks achieved final validation accuracy 73%, and as our submission to PlantCLEF 2017 ranked 3rd with Mean Reciprocal Rank 84.3%.

## Discussion

The accuracy of Ffirst is suitable for practical applications in leaf and bark recognition, exceeding 99% for most leaf datasets. The method is computationally efficient and fast: processing 200 × 200 pixel images takes about 0.05 s on a laptop without using a GPU. That makes real-time processing on common handheld devices (such as low-end smartphones) feasible. The drawback of such global texture descriptor is its dependence on perfect segmentation of the area of interest, which makes it unsuitable for more complex pictures of plants. In the case where the whole image area contains bark texture, no segmentation is needed. For leaf scans or photographs of leaves on a white background, segmentation is trivial and all information is visible in the image. For more complex cases, such as unconstrained plant recognition “in the wild” including occlusions, complex background and highly variable image content, a more generalizing model is needed.

The generality and higher capacity of CNNs is suitable for such more complex tasks. With large amounts of training data, state-of-the-art convolutional neural network architectures achieve the best results on such tasks, as validated by results of the recent PlantCLEF challenges [38, 43].

CNN models usually need a very high amount of training data for training. This need can be partially reduced by initializing the model variables from a pre-trained model (usually on ImageNet). An experiment with the modified state-of-the-art Inception-ResNet-v2 network shows that with sufficient training data, fine-tuning a deep convolutional neural network leads to almost perfect leaf classification, achieving at least 99.9% accuracy on the MEW leaf dataset. Although this leaf dataset represents a considerable number of classes (153), it is still much lower than in the case of PlantCLEF challenges (10,000 species in 2017). There is a lack of larger bark datasets for similar experiments. It is common for the more constrained tasks, that many of the publicly available datasets are rather small in the number of classes and images - the AFF datasets are a great example. This dataset size variance has to be taken into account when interpreting the achieved accuracy: for example, Ffirst achieves 100 % accuracy on the AFF leaf dataset, which only contains 5 plant species, while the 99.5% accuracy on the MEW daraset with 153 classes is definitely more informative. Besides dataset size, we also noticed a significant effect of segmentation errors on the performance in the case of the Leafsnap dataset.

The disadvantage of common CNNs are high hardware demands for training the models and for real-time processing—in practice, this is achieved by massive parallelization on GPUs or other deep-learning-specialized hardware units, such as the recently introduced Tensor Processor Units. From the network design point of view, the processing speed might be increased by quantization and pruning, but also using smaller models, such as MobileNets [93]. All of these methods, however, tend to decrease the model accuracy.

We observe that building an ensemble of such networks improves accuracy significantly by combining the expertise learned by several models converging into different local minima. We believe that this raises an interesting question for future research: How to combine ensembles of such models in a more efficient way?

## Conclusions

Identification of plant species from pictures of bark and leaves using textural recognition with the proposed *Ffirst* method leads to state-of-the-art results, while keeping computational demands small, which makes it suitable for real-time processing. Our experiment shows that with enough training data, an even better accuracy can be achieved using a convolutional neural network, performing leaf classification almost perfectly with 99.9–100.0% accuracy on the MEW dataset with 153 plant species.

The results suggest that with sufficient amount of training data, recognition of segmented leaves is practically a solved problem. Learning from a small number of samples may be still a valid problem and may be practical for uncommon plant species or rare phenotypes.

The generality and higher capacity of state-of-the-art CNNs makes them suitable for plant recognition “in the wild”, where the views on plant organs or plants vary significantly and suffer from occlusions and background clutter. That was demonstrated by the results of the recent PlantCLEF challenges [38, 43], where the proposed deep learning methods performed competitively, finishing among the top 3 teams in both 2016 and 2017.

## Abbreviations

AFF: Austrian Federal Forest (dataset)
CNN: convolutional neural network
COCO: common objects in context (dataset, challenge)
DFT: discrete Fourier transform
EoL: encyclopedia of life (web encyclopedia), http://eol.org/
FC: fully connected (layer)
Ffirst: fast features invariant to rotation and scale of texture
GPU: graphics processing unit
LBP: Local Binary Patterns
mAP: mean average precision
MEW: Middle European Woods (dataset)
SIFT: Scale Invariant Feature Transform
SVM: Support Vector Machine

## Footnotes

1
http://leafsnap.com/.

2 LBP-HF (as well as $\hbox {LBP}^{ri}$) are rotation invariant only in the sense of a circular bit-wise shift, e.g. rotation by multiples $22.5^{\circ }$ for $\hbox {LBP}_{16,R}$.

3 The Gaussian filtering is used for a scale *i* only if $\sigma _i > 0.6$, as filtering with lower $\sigma _i$ leads to significant loss of information.

4 The Computer Vision Lab, TU Vienna, kindly made the dataset available to us for academic purposes, with courtesy by Österreichische Bundesforste/Archiv.

5 In [20], the result presented as “95.44% (1820 / 1907)” seems to be tested on all images.
